# Different Molecular Interaction between Collagen and α- or β-Chitin in Mechanically Improved Electrospun Composite

**DOI:** 10.3390/md17060318

**Published:** 2019-05-30

**Authors:** Hyunwoo Moon, Seunghwan Choy, Yeonju Park, Young Mee Jung, Jun Mo Koo, Dong Soo Hwang

**Affiliations:** 1 Division of Environmental Science and Engineering, Pohang University of Science and Technology (POSTECH), 77 Chengam-ro, Nam-gu, Pohang 37673, Korea; lbemmoon@postech.ac.kr; 2 Division of Integrative Biosciences and Biotechnology, Pohang University of Science and Technology (POSTECH), 77 Chengam-ro, Nam-gu, Pohang 37673, Korea; lbemchoi@postech.ac.kr; 3 Department of Chemistry, Institute for Molecular Science and Fusion Technology, Kangwon National University, Chuncheon 24341, Korea; yeonju4453@kangwon.ac.kr (Y.P.); ymjung@kangwon.ac.kr (Y.M.J.); 4 Department of Fibre and Polymer Technology, KTH Royal Institute of Technology, Teknikringen 58, SE-100 44 Stockholm, Sweden

**Keywords:** chitin, collagen, electrospinning, mechanical property, 2D correlation spectroscopy, polymorph

## Abstract

Although collagens from vertebrates are mainly used in regenerative medicine, the most elusive issue in the collagen-based biomedical scaffolds is its insufficient mechanical strength. To solve this problem, electrospun collagen composites with chitins were prepared and molecular interactions which are the cause of the mechanical improvement in the composites were investigated by two-dimensional correlation spectroscopy (2DCOS). The electrospun collagen is composed of two kinds of polymorphs, α- and β-chitin, showing different mechanical enhancement and molecular interactions due to different inherent configurations in the crystal structure, resulting in solvent and polymer susceptibility. The collagen/α-chitin has two distinctive phases in the composite, but β-chitin composite has a relatively homogeneous phase. The β-chitin composite showed better tensile strength with ~41% and ~14% higher strength compared to collagen and α-chitin composites, respectively, due to a favorable secondary interaction, i.e., inter- rather than intra-molecular hydrogen bonds. The revealed molecular interaction indicates that β-chitin prefers to form inter-molecular hydrogen bonds with collagen by rearranging their uncrumpled crystalline regions, unlike α-chitin.

## 1. Introduction

Collagen is one of the most popular materials used as a tissue scaffold, drug carrier, cosmetic ingredient, bio-ink, dentistry and guide membrane in regenerative medicine and has been significantly utilized in medical fields [1,2,3,4,5]. Collagen mainly consists of repeating units of triplet sequence [Gly–Xaa–Yaa] with a hierarchically assembled triple helix and forms matrices of most of the vertebrate organs [6,7]. Collagen xenografts that are generally extracted from bovine or pig are non-cytotoxic, biocompatible, and easily resorbable in the human body; which is why clinicians prefer using collagen for human tissue regeneration [8,9]. However, the collagen xenografts have inherently lower mechanical strength than the native collagen tissues in humans with the hierarchical structure reinforced by Ca-based biomineralization [10,11]. 

To overcome the mechanical weakness of the collagen xenograft, without the cost of the fibrous feature, researchers have been trying to make collagen composites with varied synthetic and natural polymers including polycaprolactone, polylactic acid, polyvinyl alcohol, silk fibroin, chitosan, cellulose, and chitin [12,13,14,15,16]. Among them, chitin is the second most abundant natural polymer on Earth and can be acquired from marine creatures such as crab, shrimp, and squid. In addition, chitin has several attractive bioactivities, including environmental friendliness, biocompatibility, antibacterial activity, cost-effectiveness, and stiffness [17,18]. However, practical applications of chitin as a reinforcing material for mechanically weak polymers, such as collagen, have been impeded due to its insolubility in common solvent systems [19]. A highly crystalline structure which results from tight chain arrangement with multiple hydrogen bonds in chitin [20], limits its solubility in common organic solvent systems, thus only relatively low molecular weight and low concentrations of chitin could be dissolved in hexafluoro-2-propanol (HFIP). HFIP is a well-established halogenated solvent for electrospinning by dissolving the internal structure of polymers. However, in the case of chitin, HFIP is not a good solvent because strong hydrogen bonds in chitin prevent HFIP from infiltrating into its structure. However, it was suggested that chitin is electrospinable in acidic solvents, such as formic acid, methanesulfonic acid, basic lithium chloride (LiCl)/dimethylacetamide (DMAc), or ionic liquids [21,22]. However, only a small amount of chitin (<1%) is soluble in the above solvent systems. 

In addition, the type of chitin resource that can be utilized for making the composite has been overlooked. Chitin has two naturally occurring polymorphs that are in the α and β phase. Two different phases occur by different inter- and intra-hydrogen bonds from a myriad of hydroxyl and amide groups, resulting in α- and β-chitin having different solvent susceptibilities. Therefore, it is a prerequisite to explore which solvent system can be used for dissolving both the chitins and collagen to make a mechanically reinforced composite material. Furthermore, we investigated which chitin phase will form inter-chain interactions with collagen which are preferential for the resultant mechanical prowess. Until now, molecular interactions that determine the mechanical properties have scarcely been investigated for a chitin adjuvant.

The aim of this work is exploring the solvent system that dissolves both the chitins as well as collagen and unraveling different molecular interactions for mechanical improvement. We electrospun the composite of chitins and collagen and investigated which chitin phase is more effective to improve the mechanical stability of the collagen and further estimated the molecular interactions behind the improvement.

## 2. Results and Discussion

**Figure 1:**
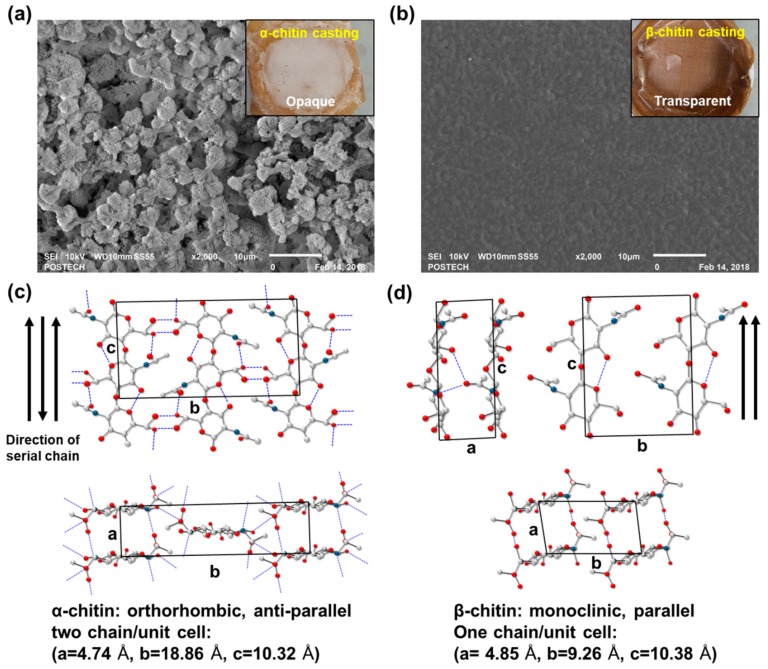
Scanning electron microscopy (SEM) micrographs of cast (**a**) α-chitin (opaque) and (**b**) β-chitin (transparent) film for different solvent susceptibilities to hexafluoro-2-propanol (HFIP)/ trifluoroacetic acid (TFA) solvent systems (magnification: 2000×). Different chain arrangements and crystal structures of (**c**) α-chitin and (**d**) β-chitin [18].

Relatively high molecular weights of α- and β-chitin were adopted as an adjuvant for generating collagen composites to investigate the different molecular interactions. Depending on the intrinsic crystal structure and inter- and intra-chain hydrogen bonds, chitins showed different solvent susceptibilities as well as blending properties. Before blending with collagen, an appropriate solvent ratio for chitins was found by an α-chitin dissolution test at various ratios between HFIP and trifluoroacetic acid (TFA) (Figure S1). We found that the combination of a halogenated solvent with a small amount of acidic solvent is effective to dissolve chitin. In the HFIP/TFA (85:15, *v*/*v*) chitin was dissolved at a maximum concentration of 5% (*w*/*v*). In Figure 1, α- and β-chitin that had been dissolved in HFIP/TFA (85:15, *v*/*v*) showed distinct film morphologies which were cast on Teflon-covered dishes in the same conditions. Interestingly, the cast film of α-chitin was opaque and had broccoli-like grain structures (~5 μm), as observed in the scanning electron microscopy (SEM) image (Figure 1a). On the contrary, β-chitin formed a relatively unstructured smooth surface, resulting in a transparent film (Figure 1b). This means that different chain arrangements and crystal structures derived from different hydrogen bond configurations affect the solvation properties and reconstructing factors. Generally, α-chitin has an orthorhombic crystal structure with unit cell dimensions of a = 4.74 Å, b = 18.86 Å, and c = 10.32 Å from the anti-parallel chain configuration and tight hydrogen bonds [18] (Figure 1c). However, a relatively loose configuration by the parallel assembly of β-chitin with a particularly short inter-plane distance, b, equal to 9.26 Å, compared to that of α-chitin makes a monoclinic crystal structure. Consequently, we hypothesized that the collagen/chitin composite probably exhibits different fiber formations and macroscopic mechanical properties depending on the type of chitin added to the composite.

**Figure 2:**
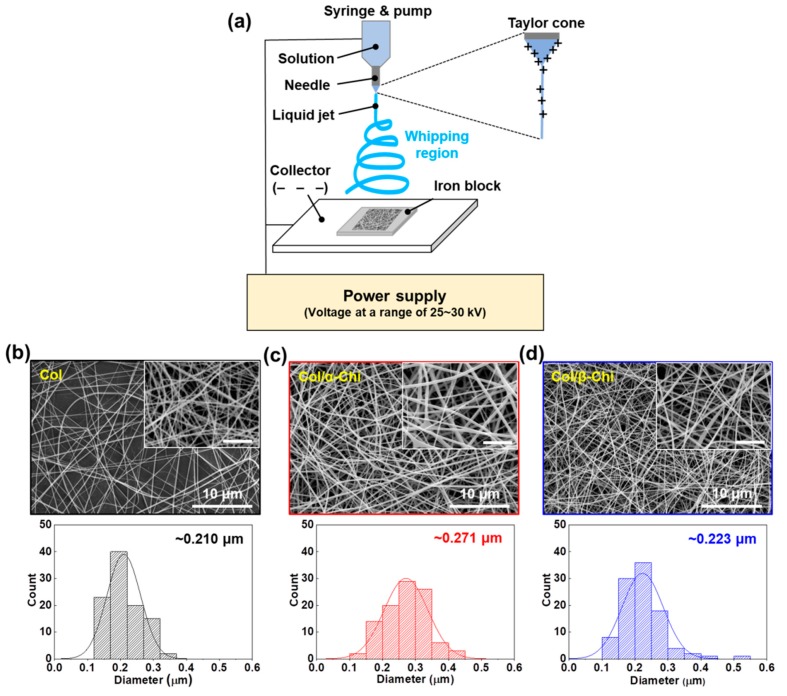
(**a**) Schematic illustration for electrospinning on iron block in HFIP/TFA (85:15, *v*/*v*). SEM observation of electrospun (**b**) Col, (**c**) Col/α-Chi, and (**d**) Col/β-Chi mats and corresponding width distributions constituting the nanofibers (n = 100, inset: magnified images and all scale bars are 3 μm).

To confirm the macroscopic mechanical effect achieved by adding the chitins into collagen, electrospinning was utilized to obtain well-blended composites [23]. For the mat formation, relevant electrospinning conditions were evaluated in terms of the voltage, pumping speed, and needle tip-to-collector distance. A custom-made iron block was placed on the collector to harvest homogeneously made mats, as illustrated in Figure 2a. The tip-to-plate distance was set at 8 cm, otherwise, the fiber would snap (>8 cm) or it would be too difficult to form fiber, due to lack of the solvent vaporization (<8 cm). Furthermore, optimized electrospinning conditions of applied voltage and flow rate were 25–30 kV and 0.25 mL/h, respectively. The concentration of chitin and collagen was set to 1% (*w*/*v*) and 13% (*w*/*v*), respectively, for electrospinability. As a result, we successfully made three kinds of mats—collagen (Col), collagen/α-chitin (Col/α-Chi), and collagen/β-chitin (Col/β-Chi). The Col composed of collagen nanofibers which had a fiber diameter of ~0.210 μm (Figure 2b). However, changes in the diameter of the nanofiber by adding different chitins showed a different appearance, which can be determined by the pattern of molecular interaction, including molecular distance, the nanocrystalline structure, and distribution of the crystalline phase [24]. The Col/α-Chi showed a diameter that was ~29% greater than that of Col (~0.271 μm) (Figure 2c). This increase was due to the added crystalline phase α-chitin, which probably indicates that inhomogeneous blending of collagen and α-chitin occurred. On the other hand, nanofibers in Col/β-Chi showed a similar diameter to that of Col, achieving a slight increase to ~0.223 μm.

**Figure 3:**
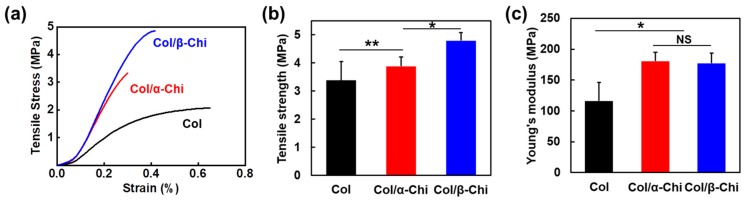
Mechanical improvement of Col by adding α- and β-chitin. (**a**) Representative stress–strain curves of electrospun mats during uniaxial tensile test. Statistical evaluation of (**b**) tensile strength and (**c**) Young’s modulus (the mean value ± SD; n = 6, * *p* < 0.05; ** *p* < 0.01; NS, not significant).

To reveal how the blending characteristics at the molecular level and interactions between collagen and chitin affect the macroscopic mechanical properties, electrospun mats were subjected to a uniaxial tensile test (Figure 3a). The tensile strength was increased from 3.4 ± 0.7 to 3.9 ± 0.3 and 4.8 ± 0.3 MPa for Col/α- and Col/β-Chi, respectively (Figure 3b). As expected, Col/β-Chi showed better tensile strength, with a value that was ~41% and ~14% greater than Col and Col/α-Chi, respectively, due to a favorable secondary interaction, i.e., intermolecular hydrogen bonds. This could be explained by homogeneous crystal dissolution and the formation of new hydrogen bonds. Furthermore, increased uncrumpled regions of β-chitin enable more collagen–chitin interactions than intra-molecular interactions within the chitin. It manifests as an effective energy dissipation by collaborating between collagen and chitin despite the loss of the crystalline phase of β-chitin. However, Young’s modulus of Col/β-Chi (177.3 ± 16.6 MPa) was comparable to that of Col/α-Chi (180.6 ± 14.07 MPa), even though both the mats were stiffer than Col (116.2 ± 0.7 MPa) (Figure 1c). We anticipate that these interesting features are probably due to solvent susceptibility, which could originate from the inherently developed chain arrangement and the resultant crystal structure.

To understand the mechanical behavior derived from α- and β-chitin, it is important to re-evaluate the structural properties of chitins and collagen. As mentioned, chitin has two major naturally occurring crystalline polymorphs, α- and β-chitin. The proposed unit cell for α-chitin is orthorhombic with an antiparallel arrangement [25]. This structure allows α-chitin to have a three-dimensional cooperative hydrogen bond network that includes inter-sheet hydrogen bonds, making it thermodynamically more stable than β-chitin [26]. The unit cell of β-chitin is monoclinic with a parallel chain arrangement. It consists of well-defined sheets but lacks hydrogen bonding. Such an anisotropic nature and weak association of the sheets leads to an unstable state, which results in the superior susceptibility to other materials compared to the susceptibility of α-chitin [27,28]. In detail, there are three major hydrogen bonds, two intermolecular and one intramolecular, that are not cooperative. Unlike α-chitin, unoccupied hydrogen-bonding acceptors in β-chitin are isolated, which act as hydrogen bonding terminators [29]. 

Meanwhile, collagen mainly consisting of glycine, proline, and hydroxyproline are structured as a supercoil intertwined with triple helices. Unlike a globular structure, the close association of three chains leaves no room for interior spaces or cavities in the triple helix [30]. It protrudes polar domains/side chains, such as carbonyl (C=O) and amine (N–H) functional groups, to the surface of the fibrous structure and exposes them for interaction. In theory, β-chitin with susceptible structural characteristics could form a complex through extensive hydrogen bonding on the surface of fibrous collagen. The mechanical result of incorporating chitin to collagen supports such a theory. This can be seen in Figure 3b, Col/β-Chi showed a tensile strength of 4.8 MPa that is 14% higher than that of Col/α-Chi.

**Figure 4:**
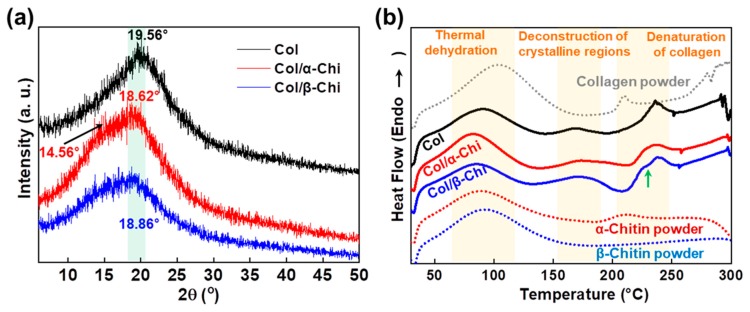
(**a**) Changes in the X-ray diffraction pattern and (**b**) differential scanning calorimetry depending on the corresponding chitin adjuvants (each powder indicates raw materials without electrospinning).

To determine the effect of α- and β-chitin on the structure of collagen, XRD and thermal analysis were conducted. As presented in Figure 4a, the peak position of collagen at 19.56° shifted to 18.62° and 18.86° corresponding to α- and β-chitin, respectively [31,32,33]. A decrease in the 2θ value implies that chitin polymorphs are able to penetrate and position themselves in the space between collagen fibers, facilitated by the strong dissolution effect of TFA [34]. However, there was a noticeable shoulder peak at 14.56° in α-chitin, which signifies that there are two distinctive phases in the composite, originating from the lack of any secondary interaction between α-chitin and collagen. It is also interesting to note that the peak intensity of Col/β-Chi is approximately half that of collagen or Col/α-Chi. This means that the structural integrity of collagen has significantly decreased with the addition of β-chitin by the formation of new secondary interactions. This observation supports the result of mechanical properties where the formation of new hydrogen bonds between β-chitin and collagen is stronger than the intermolecular interaction between collagens. Additional evidence can be obtained from the thermal behavior. In the dehydration region, ∆H of Col, Col/α-Chi, and Col/β-Chi corresponds to ~184.54, ~215.02, and ~142.03 J/g, respectively. The energy required for thermal dehydration can imply the hydrogen bonding state of collagen and chitin. As expected, Col/α-Chi shows the highest ∆H since the sites (acceptor and donor) from collagen and α-chitin would not interact and would be occupied by water. A low ∆H value of Col/β-Chi emphasizes that hydrogen bonding sites occupied by water are replaced by a new interaction formed between β-chitin and collagen. The temperatures at which the deconstruction of the crystalline region for Col, Col/α-Chi, and Col/β-Chi begin are ~145 °C, ~139 °C, and ~132 °C, respectively. Since the structural integrity of collagen is heavily affected by the addition of β-chitin, diverse crystalline structures and sizes are present in the system. Thus, the regional area is extended while the starting point for crystal deconstruction shows the lowest value, ~132 °C. Further evidence of new secondary interactions can be observed in the denaturation region of collagen. As indicated with the green arrow at ~229 °C, Col/β-Chi showed a distinctive shoulder in this region followed by a peak at a temperature of ~237 °C, identical to collagen. This shoulder peak is an indication that a new structural interaction between β-chitin and collagen has been formed.

**Figure 5:**
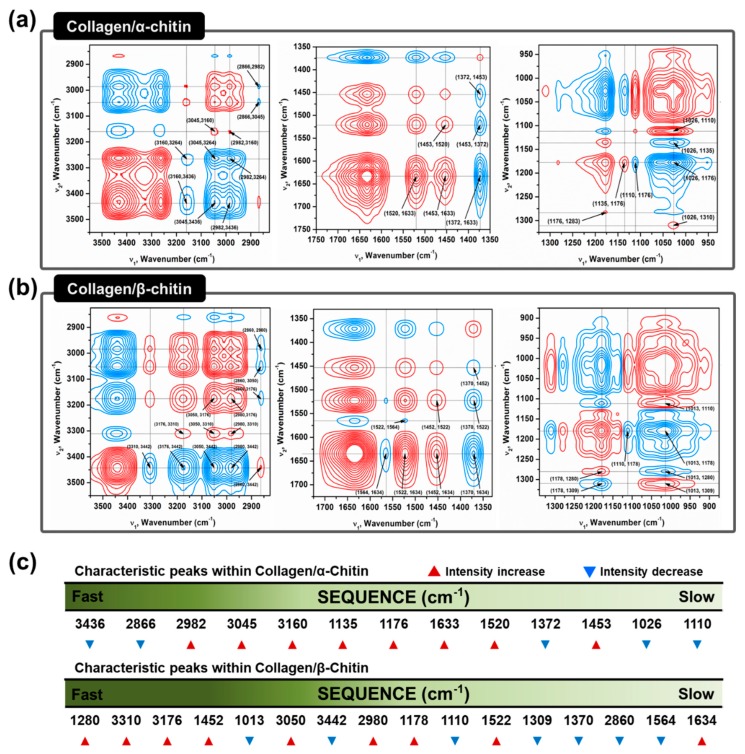
Two-dimensional correlation spectroscopy (2DCOS) analysis to confirm different molecular interactions between collagen and chitins in an HFIP/TFA system. A synchronous contour map from concentration perturbation of (**a**) α-chitin and (**b**) β-chitin in the collagen composites. (**c**) Relative interaction sequence and peak intensity variation of characteristic peaks from concentration perturbation.

The absence of a secondary interaction by α-chitin and additional hydrogen bonding formation by β-chitin in the collagen composites are visualized using two-dimensional correlation spectroscopy (2DCOS). 2DCOS is one of the most powerful and versatile spectral analysis methods that can highlight the perturbation factors, such as time, temperature, concentration, or pressure. The result of such perturbations applied to the system transforms the behavior of the chemical constituents that can be produced into a simplified result [35]. Synchronous and asynchronous 2DCOS obtained from FT–IR spectra of collagen/α- and β-chitin with increasing chitin contents identify the effect on the collagen structure. Since the intensity of the FT–IR peak is heavily dependent on the concentration of the specific functional group, an increasing or decreasing intensity with increasing chitin content would reveal the presence of a new interaction between them. With the exception of diagonal positive autopeaks (a result of the autocorrelation function of intensity variation) in synchronous 2DCOS, the positive cross peak (red) represents the intensity of the two bands changing in the same direction, either increasing or decreasing together, whereas the negative cross peaks (blue) indicates that one band is increasing as the other is decreasing. When designated cross-peaks from the synchronous 2DCOS match the sign of the asynchronous 2DCOS, the intensity on the x-axis occurs before that of the y-axis and vice versa. Given this information, 2DCOS was conducted and the result is presented in Figure 5a,b with all the peak assignments summarized in Tables S1 and S2. The identical peaks that represent the basic functional groups of chitins and collagens are at 3160–3176, 3045–3050, 1633–1634, 1520–1522, 1370–1372, 1176, and 1110 cm^-1^, which share identical increases/decreases in intensity [36,37] (Figure 5c). Moreover, C–H related peaks such as 2982 and 2866 cm^-1^, are not considered due to a lack of information. Without these groups of peaks, the remaining information from the sequence clearly represents the difference in the interactive behavior between α/β-chitin and collagen. Initial impressions of the sequence reveal that there are higher wavenumbers of peaks in collagen/β-chitin 2DCOS sequences compared to collagen/α-chitin. This alone passively informs that collagen is more interactive and sensitive to the concentration of β-chitin than to α-chitin. The peaks at 3442 and 1013 cm^−1^, corresponding to free O–H and chitin C=O, respectively, function as hydrogen-bonding acceptors and these are only present in collagen/β-chitin 2DCOS sequences [38,39]. The decrease in the intensity indicates that the number of acceptors is decreasing with the formation of new hydrogen bonds with collagen. Additionally, the initial stage of collagen/β-chitin sequence (1280, 3310, and 3176 cm^−1^ representing N–H) are donor-focused and the intensity increases, meaning that the addition of β-chitin creates a donor-friendly environment that increases the chance of new hydrogen bonding formations since characteristically, β-chitin has an abundance of acceptors. Additionally, the peak at 3436 cm^−1^, which represents intramolecular hydrogen bonding of α-chitin is at the start of the collagen/α-chitin 2DCOS sequence. This means that as the concentration of added chitin increases, α-chitin loses its ability for intramolecular hydrogen bonding.

## 3. Conclusions

In summary, we prepared electrospun collagen composites with α-chitin and β-chitin to improve the mechanical properties of the collagen. β-chitin/collagen composites showed better mechanical properties than those of α-chitin/collagen composites. 2DCOS analysis indicates that β-chitin prefers to form intermolecular hydrogen bonds with collagen by rearranging its crystalline regions, which contributes to the increase in the mechanical properties. Consequently, we suggested a means to make the composite through which the chitin phase will achieve a mechanical improvement of collagen.

## 4. Materials and Methods

### 4.1. Materials

Type I collagen from porcine skin (PSP-141706, Mw ~30,000) was purchased from SK Bioland Co., Ltd (Cheonan, Korea). High-purity α-chitin (from shrimp, C9752) and β-chitin (from squid pen, ARA-170124FC) were purchased from Sigma-Aldrich and AraBio Co., Ltd. (Seoul, Korea), respectively. The unit cell of α-chitin is a = 4.74 Å, b = 18.86 Å, and c = 10.32 Å, whereas the β-chitin is structurally different, with a = 4.85 Å, b = 9.26 Å, and c = 10.38 Å. In addition, different 2θ values of α- and β-chitin were 9.1 and 7.9 ° for (020); 20 and 19.6 ° for (110), respectively [40,41]. Hexafluoro-2-propanol (HFIP, A1247, Alfa Aesar) and trifluoroacetic acid (TFA, L06374, Alfa Aesar) were used to dissolve collagen and the two types of chitins.

### 4.2. Electrospinning

Collagen, α-chitin, β-chitin, and their blends were dissolved in HFIP/TFA (85:15, *v*/*v*) solvent at a concentration of 14% (*w*/*v*). The collagen/chitin blend was prepared in a ratio of 13:1. For the complete dissolution of chitin, the corresponding amount of chitin was first dissolved in the solvent in a 40 °C oven for 24 h, followed by the addition of collagen for another 12 h. Pure collagen for use as a control was dissolved in the solvent for 12 h at a concentration of 14% (*w*/*v*). Electrospinning was then performed with a customized electrospinning machine (Nano NC, Seoul, Korea) and syringe pump (KDS100, KD Scientific, Holliston, MA, USA). The solution was placed in a 1 mL syringe with a 26-gauge needle with a clamp connected to serve as an anode with a collector to serve as the cathode, both of which were connected to a high-voltage power supply. The iron block made of 2 × 2 × 1 cm was used as the collector. The distance between the needle edge from the polymer jet and the collector was maintained at 8 cm. The polymer jets generated from the needle by the strong voltage formed ultra-fine nanofibers that accumulated down at the collector in the form of a membrane. The applied voltage and solution feed rate was adjusted to 30 kV and 0.25 mL/h, respectively. All the experiments were carried out at room temperature under 40 ± 2% relative humidity. All the membrane samples were vacuum dried in a 40 °C oven overnight to remove any solvents which had remained prior to characterization.

### 4.3. Scanning Electron Microscopy 

SEM (JSM-6010LV, JEOL, Tokyo, Japan) was used to observe the morphology of the electrospun nanofiber at an accelerating voltage of 10 kV. Sputter coating is performed for 100 s using a gold coating machine (108 Auto Sputter Coater, Cressington Scientific Instruments Co., Ltd., Watford, UK) to observe the nanofibers. Based on the SEM photographs, the average diameter of the nanofibers was analyzed using the Image J software (Wayne Rasband, National Institutes of Health, USA, http://imagej.nih.gov/ij).

### 4.4. Fourier Transform—Infrared Spectroscopy 

A Fourier transform-infrared spectroscopy (FT–IR) spectrophotometer (Nicolet™ iS™ 50 FT–IR Spectrometer, Thermo Scientific Co., Ltd., Waltham, MA, USA) was used for FT–IR analysis and measurements were carried out in the range of 4000 to 400 cm^−1^ by using the absorption mode. To observe the interaction between collagen and chitin, the concentration of collagen and chitin was adjusted to a 13:1, 12:2, 11:3, 10:4, and 9:5 ratio, and the concentration of blends was adjusted to 14% (*w*/*v*) in a HFIP/TFA (*v*/*v*, 85/15) solution. The polymer solution was poured onto a silicon wafer and cast for more than 24 h. The vibration modes of each specimen were then recorded for 2DCOS. 

### 4.5. 2DCOS Analysis

2DCOS was used to differentiate between the effects of α- and β-chitin on collagen by setting the content of chitin as a perturbation factor. Synchronous and asynchronous 2D correlation spectra were obtained using the 2DShige program (freely downloadable software developed by Prof. Shigeaki Morita (Osaka Electro-Communication University, Japan)), where the red and blue lines in the 2D correlation spectra represent positive and negative cross peaks, respectively.

### 4.6. Differential Scanning Calorimetry 

Differential Scanning Calorimetry (DSC) (DSC 4000, Perkin Elmer, Waltham, MA, USA) was used to observe the thermal behaviors of the electrospun mat. Five milligrams of each specimen were heated at a scan rate of 10 °C/min from 30 to 300 °C under nitrogen gas conditions.

### 4.7. X-ray Diffraction 

Crystal X-ray analysis was taken with Ni-filtered CuKα radiation (λ = 1.5418 Å) using a D/MAX-2500/PC instrument (Rigaku, Tokyo, Japan) under 40 kV/100 mA. The diffractogram was recorded in an angular range of 5° to 60° (2θ) with a step size of 0.02° and a scanning speed of 2° min^−1^.

### 4.8. Uniaxial Tensile Test

Mechanical properties of the electrospun collagen/chitin mats were applied to a universal testing machine (UTM, 3344, Instron, Norwood, MA, USA) for confirmation of the reinforced mechanical properties. It was equipped with a 2 kN load cell and a 1 mN resolution with ±0.5% uncertainty. All samples were cut into 5 mm × 20 mm sizes, and the portion held by the jig was 5 mm in the up and down direction, and the length of the extended portion was 10 mm. The tensile speed was adjusted to 10 mm/min. The tensile test was carried out at room temperature with a relative humidity of 40 ± 2%. The thickness was measured using a Digimatic Micrometer (293–240, Mitutoyo, Kawasaki-shi, Japan) using the values averaged by five points for each sample.

### 4.9. Statistical Analysis

All of the experimental data were presented as the mean ± standard deviation (SD) and were performed at least three independent times. The differences between the experimental data were examined using a student’s t-test. A *p*-value < 0.05 was considered to be statistically significant.

## Supplementary Materials

The following are available online at https://www.mdpi.com/1660-3397/17/6/318/s1, Figure S1: Solubility test, Figure S2: Heterogeneous and asynchronous contour map of 2DCOS, Table S1–S2: Results of 2DCOS, Table S3: DSC peak assignment and calculated ΔH values.
