# Supplementary material for: Different Molecular Interaction between Collagen and α- or β-Chitin in Mechanically Improved Electrospun Composite
Source: Mar Drugs. 2019 May 30;17(6):318. doi: 10.3390/md17060318 (PMC6628339; doi:10.3390/md17060318)
Supplement: Supplementary file 1 [file marinedrugs-17-00318-s001.pdf]

marine drugs

Supplementary Materials

Different Molecular Interaction between Collagen and α- or β-Chitin in Mechanically Improved Electrospun Composite

Hyunwoo Moon[1,#], Seunghwan Choy[1,#], Yeonju Park[2], Young Mee Jung[2], Jun Mo Koo[3,*], Dong Soo Hwang[1,*]

[1] Department of Integrative Bioscience and Biotechnology, Pohang University of Science and Technology (POSTECH), 77 Chengam-ro, Nam-gu, Pohang 37673, Korea

[2] Department of Chemistry, Institute for Molecular Science and Fusion Technology, Kangwon National University, Chuncheon 24341, Korea

[3] Department of Fibre and Polymer Technology, KTH Royal Institute of Technology, Teknikringen 58, SE-100 44, Stockholm, Sweden

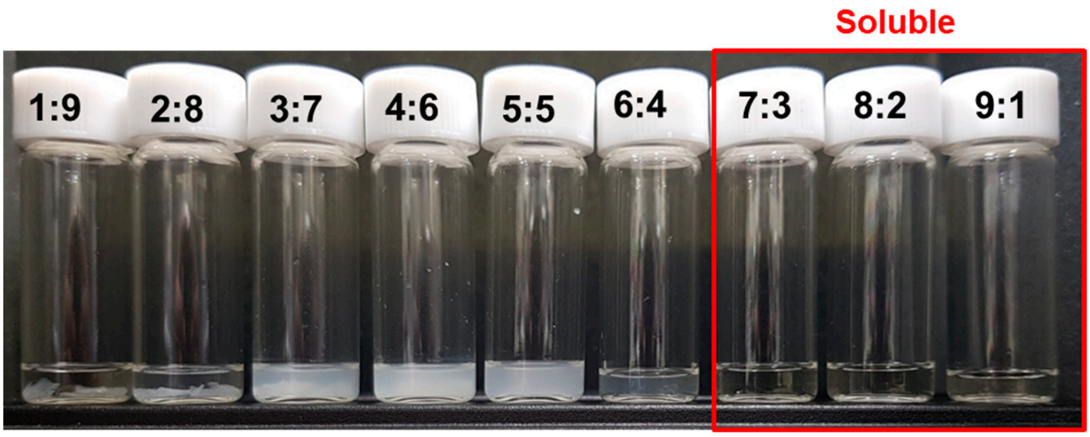

Figure S1. Representatively conducted solubility test of α-chitin in various ratio of HFIP:TFA solvent (all the solution concentrations were 1% (w/v)). Pure HFIP and TFA were excluded owing to its extremely poor solubility on chitin.

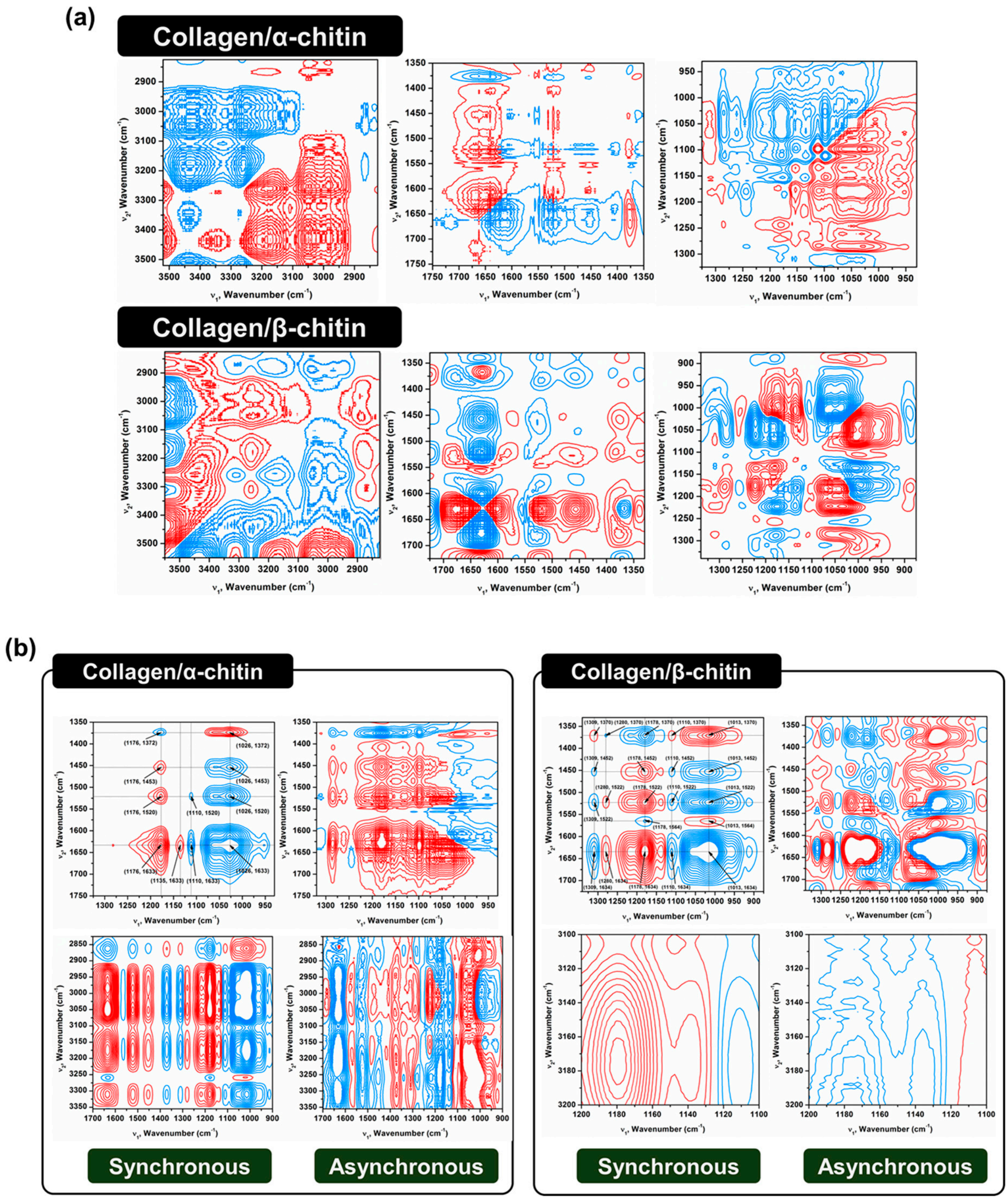

Figure S2. (a) Asynchronous 2DCOS and (b) heterogeneous contour map.

Table S1. 2DCOS analysis between collagen and α-chitin and corresponding sequences and intensity changes.

Sequential order	Wavenumber (cm^{-1})	Intensity change	Assignment	Origin	Reference
1	3436	▼	Intramolecular hydrogen bonding (O–H)	Chitin	[6,9]
2	2866	▼	-CH3	Collagen	[2,3]
3	2982	▲	-CH2	Chitin	[9]
4	3045	▲	N–H	Chitin	[9]
5	3160	▲	N–H	Chitin	[9]
6	1135	▲	C–O–C	Collagen, chitin	[2,3,9]
7	1176	▲	C–O	Collagen	[5]
8	1633	▲	C=O	Collagen, chitin	[1-3,8,10]
9	1520	▲	Amide N–H	Collagen, chitin	[2,5,9]
10	1372	▼	C–H, CH3	Collagen	[1]
11	1453	▲	CH2	Chitin	[1,4,5]
12	1026	▼	C–O	Collagen, chitin	[7,9]
13	1110	▼	C=O, C–O	Chitin	[8,9]

Table S2. 2DCOS analysis between collagen and β-chitin and corresponding sequences and intensity changes.

Sequential order	Wavenumber (cm^{-1})	Intensity change	Assignment	Origin	Reference
1	1280	▲	C–N, N–H	Collagen, chitin	[1-5,8,9]
2	3310	▲	N–H	Collagen	[2,6]
3	3176	▲	N–H	Chitin	[11,9]
4	1452	▲	Amide N–H	Collagen, chitin	[2,5,8,9]
5	1013	▼	C=O	Chitin	[8]
6	3050	▲	N–H	Chitin	[9,11]
7	3442	▼	OH	Collagen, chitin	[2,6,9]
8	2980	▲	CH3	Chitin	[9]
9	1178	▲	C–O	Collagen	[5]
10	1110	▼	C=O, C–O	Chitin	[8,9]
11	1522	▲	N–H	Collagen, chitin	[2,5,9]
12	1309	▼	CH2	Collagen	[7]
13	1370	▼	C–H, CH3	Collagen	[2]
14	2860	▼	-CH3	Collagen	[3]
15	1564	▼	Amide N–H	Collagen, chitin	[5,9]
16	1634	▲	Amide C=O	Collagen, chitin	[2,5,8]

Table S3. The results of differential scanning calorimetry (DSC) analysis.

Specimen	Temperature (°C)			ΔH (J·g^{-1})	State	Reference
	Onset	Peak	End			
Collagen	37	104	179	369.59	Dehydration	[12,13]
α-Chitin	39	88	184	230.45	Dehydration	
β-Chitin	39	91	180	265.97	Dehydration	
Col	37	91	143	184.54	Dehydration	
	205	235	250	20.5	Denaturation	
Col/α-Chi	37	82	139	215.02	Dehydration	
	215	235	257	27.34	Denaturation	
Col/β-Chi	37	85	133	142.03	Dehydration	
	211	237	255	39.77	Denaturation	

References

1. Belbachir, K.; Noreen, R.; Gouspillou, G.; Petibois, C. Collagen types analysis and differentiation by FTIR spectroscopy. *Anal. Bioanal. Chem.* **2009**, *395*, 829–837. https://doi.org/10.1007/s00216-009-3019-y
2. Ungureanu, C.; Ioniță, D.; Berteanu, E.; Tcacenco, L.; Zuav, A.; Demetrescu, I. Improving Natural Biopolymeric Membranes Based on Chitosan and Collagen for Biomedical Applications Introducing Silver. *J. Braz. Chem. Soc.* **2015**, *26*, 458–465. https://dx.doi.org/10.5935/0103-5053.20150298
3. Fernandes, L.L.; Resende, C.X.; Tavares, D.S.; Soares, G.A.; Castro, L.O.; Granjeiro, J.M. Cytocompatibility of Chitosan and Collagen-Chitosan Scaffolds for Tissue Engineering. *Polímeros* **2011**, *21*, 1–6. https://dx.doi.org/10.1590/S0104-14282011005000008
4. Lee, Y.-C.; Chiang, C.-C.; Huang, P.-Y.; Chung, C.-Y.; Huang, T.D.; Wang, C.-C.; Chen, C.-I.; Chang, R.-S.; Liao, C.-H.; Reisz, R.R. Evidence of preserved collagen in an Early Jurassic sauropodomorph dinosaur revealed by synchrotron FTIR microspectroscopy. *Nat. Commun.* **2017**, *8*, 14220. https://doi.org/10.1038/ncomms14220
5. Patrick, G.; Heidrun, S. Fourier-Transform Midinfrared Spectroscopy for Analysis and Screening of Liquid Protein Formulations. Part 2: detailed analysis and applications. *Bioprocess Int.* **2006**, *4*, 48–55
6. Satio, Y.; Iwata, T. Characterization of hydroxyl groups of highly crystalline β-chitin under static tension detected by FT-IR. *Carbohydr. Polym.* **2012**, *87*, 2154–2159. https://doi.org/ 10.1016/j.carbpol.2011.10.044
7. Veeruraj, A.; Arumugam, M.; Ajithkumar, T.; Balasubramanian, T. Isolation and characterization of collagen from the outer skin of squid (*Doryteuthis singhalensis*). *Food Hydrocoll.* **2015**, *43*, 708–716. https://doi.org/10.1016/j.foodhyd.2014.07.025
8. Rahman, M.A.; Halfar, J. First evidence of chitin in calcified coralline algae: new insights into the calcification process of Clathromorphum compactum. *Sci. Rep.* **2014**, *4*, 6162. https:// doi.org/10.1038/srep06162
9. Ca´rdenas, G.; Cabrera, G.; Taboada, E.; Miranda, S.P. Chitin Characterization by SEM, FTIR, XRD, and 13C Cross Polarization/Mass Angle Spinning NMR. *J. Appl. Polym. Sci.* **2004**, *93*, 1876–1885. https://doi.org/10.1002/app.20647
10. Jang, M.-K.; Kong, B.-G.; Jeong, Y.I.; Lee, C.H.; Nah, J.-W. Physicochemical Characterization of α-Chitin, β-Chitin, and γ-Chitin Separated from Natural Resources. *J. Polym. Sci. A Polym. Chem.* **2014**, *42*, 3423–3432. https://doi.org/10.1002/pola.20176
11. Ifuku, S.; Nogi, M.; Abe, K.; Yoshioka, M.; Morimoto, M.; Saimoto, H.; Yano, H. Preparation of Chitin Nanofibers with a Uniform Width as α-Chitin from Crab Shells. *Biomacromolecules* **2009**, *10*, 1584–1588. https://doi.org/10.1021/bm900163d
12. Bozec, L.; Odlyha, M. Thermal Denaturation Studies of Collagen by Microthermal Analysis and Atomic Force Microscopy. *Biophys. J.* **2011**, *101*, 228–236. https://doi.org/10.1016/j.bpj.2011.04.033

13. Kayaa, M.; Mujtaba, M.; Ehrlich, H.; Salaberria, A.M.; baran, T.; Amemiya, C.T.; Galli, R.; Akyuz, L.; Sargin, I.; Labidi, J. On chemistry of γ-chitin. *Carbohydr. Polym.* **2017**, *176*, 177–186. http://dx.doi.org/10.1016/j.carbpol.2017.08.076

© 2019 by the authors. Licensee MDPI, Basel, Switzerland. This article is an open access article distributed under the terms and conditions of the Creative Commons Attribution (CC BY) license (http://creativecommons.org/licenses/by/4.0/).
